# A Simple and Efficient Method to Isolate LTR Sequences of Plant Retrotransposon

**DOI:** 10.1155/2014/658473

**Published:** 2014-05-06

**Authors:** Da-Long Guo, Xiao-Gai Hou, Xi Zhang

**Affiliations:** ^1^College of Forestry, Henan University of Science and Technology, Luoyang, Henan 471003, China; ^2^College of Agriculture, Henan University of Science and Technology, Luoyang 471003, China

## Abstract

Retrotransposons (RTNs) have important roles in the formation of plant genome size, structure, and evolution. Ubiquitous distributions, abundant copy numbers, high heterogeneities, and insertional polymorphisms of RTNs have made them as excellent sources for molecular markers development. However, the wide application of RTNs-based molecular markers is restricted by the scarcity of the LTR (long terminal repeat) sequences information. A new, simple, and efficient method to isolate LTR sequences of RTNs was presented based on the degenerate RNase H nested primers and PPT (polypurine tract) primer of RTNs in tree peony. This method combined the characteristics and advantages of high-efficiency thermal asymmetric interlaced PCR (hiTAIL-PCR), annealing control primer (ACP) system, and suppression PCR method. Nineteen LTR sequences were isolated using this new method in tree peony and the applicability of the LTR sequences based markers was validated by further SSAP analysis. The results showed that the new method is simple, of low-cost, and highly efficient, which is just conducted by three rounds of PCR and does not need any restriction enzymes and adapters, much less the hybridizations. This new method is rapid, economical, and cost- and time-saving, which could be easily used to isolate LTR sequences of RTNs.

## 1. Introduction

Retrotransposons (RTNs) are the most abundant and wide-distributed mobile genetic elements in eukaryotic genomes and they inserted into the host genome via an RNA intermediate [1]. RTNs can be divided into two major groups: LTR (long terminal repeat) and non-LTR retrotransposons according to the presence or absence of LTR [2]. LTR-RTNs are ubiquitous in the plant kingdom and present in high copy numbers and constitute major parts of plant genomes, which can comprise 40–90% of the genome as a whole [2]. RTNs have important roles in the formation of plant genome size, structure, and evolution [2–4]. They have been employed as an efficient tool in gene cloning, gene expression, and phylogenetic analysis [5, 6]. Ubiquitous distributions, abundant copy numbers, high heterogeneities, and insertional polymorphisms, both within and between plant LTR retrotransposons, have made them as excellent sources for molecular markers development [6, 7] and obtained more attractions than other conventional markers due to their specific characteristics [7]. The molecular markers based on the RTNs have been developed constantly, such as SSAP [8], RBIP [9], iPBS [10], IRAP, and REMAP [11], and have shown large superiority over other conventional molecular markers [6, 12, 13]. These RTN-based markers revealed insertion polymorphism between RTNs and other elements due to their different primer design principles [7]. Usually, SSAP shows more polymorphism and more codominance than AFLP [7] and it is more informative for studying genetic diversity in tomato and pepper than SSR and AFLP [12]. A major disadvantage of all the methods described above is the need for LTR sequence information of RTNs to design species-specific primers [7], especially for SSAP.

RTNs have many conserved motifs, such as sequence of reverse transcriptase (RT) [3], RNase H [14], primer binding site (PBS) [10], and polypurine tract (PPT) [15]. LTR-RTNs share a unique structural feature. Two long terminal repeats (LTRs) are longer than 100 bp and play a key role in their transposition [2]. PBS is adjacent to the 5′-LTR and used to prime the reverse transcriptase-catalyzed synthesis of minus-strand cDNA [10]. PPT is located upstream of the 3′-LTR and is a site of plus-strand synthesis initiation [2, 16]. Another important element of RTNs, RNase H, is responsible for the degradation of the RNA template in the DNA-RNA hybrid. The specific structural features of LTR-RTNs have been described by Wicker et al. [17].

Pearce et al. [14] firstly reported a novel technique for rapid isolation of plant Ty1-*copia* terminal repeat sequences of RTNs based on the consensus amino acids of RNase H motif. After that, various isolation methods of LTR-RTNs have been developed based on the conserved primers [10, 14] and genome walking methods [15, 18–21]. These methods have different principles and efficiencies, but some of them either need probe hybridization or need enzyme digestion and adapter ligation. These lead to the tedious procedures and influence the isolation efficiency. Compared with the existing methods for isolation of LTR sequence, the key factor is the efficiency of chromosome walking which is used to clone unknown LTR region based on the conserved RNase H or PPT motif of RTNs [15, 18–22].

In this study, a new, easy, fast, and efficient isolation method of RTN-LTRs was proposed and validated in tree peony. This method combined the characteristics and advantages of high-efficiency thermal asymmetric interlaced PCR (hiTAIL-PCR) [23], annealing control primer (ACP) system [24], and suppression PCR [22, 25] method. It is simple, low-cost, and highly efficient, which is just conducted through three rounds of PCR and does not need any restriction enzymes and adapters, much less the hybridizations.

## 2. Materials and Methods

### 2.1. Materials and DNA Extraction

Tree peony (*Paeonia suffruticosa* Andrews) cultivar, “Luoyanghong,” was used to isolate the LTR sequence. The fresh leaves were collected from the Luoyang National GenBank of Tree Peony, China. Total DNA was extracted from the fully expanded true leaves using the CTAB method [26].The material information used for SSAP analysis is shown in Table 1.

### 2.2. PCR Amplification

The components of PCR reaction and PCR program of three rounds were presented in Tables 2 and 3, respectively. The nested RNase H primers used in this study were according to that of Pearce et al. [14]. The ACP primers were followed as that of Hwang et al. [24]. The UP (universal primer) primer was designed based on the primers of suppression PCR [25]. PPT primer was designed to be degenerate primer based on the sequence characteristics of PPT of RTNs [16]. The corresponding primers sequences are shown in Table 4. PCR products from the third round of PCR were cloned into the PMD-18T vector using the TA Cloning Kit (TaKaRa, Dalian, China). The ligation products were transformed into DH5*α* competent cell and positive clones were sequenced by Sun Biotech Co. (Beijing, China).

### 2.3. Sequence Analysis

The nature of cloned sequences was confirmed by performing similarity searches with known RTNs sequences from other plants in NCBI database using BLASTN, BLASTX and TBLASTX algorithms with the default parameters. Genomic DNA sequences were deposited in the GenBank databases. Multiple DNA sequence alignments were carried out using Lasergene 8.0 with MegAlign module by Jotun Hein method. The LTR and PPT segments of the sequence were identified by comparing with the known structural characteristics of LTR and PPT regions of RTNs [14, 15].

### 2.4. SSAP Analysis

SSAP amplification was performed as described by Bousios et al. [19]. SSAP analysis was also carried out twice for each primer pair in order to check the consistency and reproducibility of the SSAP markers. The sequences of SSAP adapters and adapter primers were the same as Vos et al. [27]. RTN primers were designed based on the isolated LTR sequences according to the method of Waugh et al. [8]. Selective amplification was conducted with a RTNs primer in combination with either* Mse* I + 3 or* EcoR* I + 3 [27]. The resulting bands were detected using the silver staining protocol following the method of Bassam et al. [28].

## 3. Results and Discussion

### 3.1. The Primer Design Principle of the New Method

The success of PCR amplification relies on the binding specificity; that is, a primer anneals to its target sequences. Therefore, it is important to optimize this molecular interaction [29]. In order to improve the isolation efficiency of LTR sequence, the method of high-efficiency TAIL-PCR (hiTAIL-PCR) presented by Liu and Chen [23] was firstly considered, which allows efficient amplification of large unknown target sequences [23]. The longer AD (LAD) primers of hiTAIL-PCR are 33 or 34 nucleotides which contained six or seven degenerate nucleotides and four fixed nucleotides at the 3′ ends. The arbitrary 3′ four-base sites have a moderate frequency (on average 256 bp at a time) to anchor the LADs to the unknown target regions. Three nested specific primer sets based on the known sequences were used to conduct genome walking in combination with the LAD primers. The specific PCR products were amplified through the thermal asymmetric interlaced PCR programs and subsequent PCR reactions. In order to employ hiTAIL-PCR in the new method, the nested specific primers in the original hiTAIL-PCR were replaced by two nested RNase H primers [14] and one PPT degenerate primer based on the conserved motif of RNase H and PPT of RTNs. At the same time, it is a key factor for the success of PCR amplification that the primers could bind to its target sequences or not at the period of annealing. Therefore, the optimization of primer structure is very crucial for PCR amplification. The primer annealing temperature determines that the primer completely binds to the template or partially mismatches the template in one or several bases. Consequently, the adjustment of primer annealing temperature could improve the combination specificity of primers and template. Hwang et al. [24] designed the annealing control primer (ACP) in order to enhance the specificity of PCR amplification. ACP primer is composed of three parts: a polydeoxyinosine [poly(dI)] linker between the 3′ end target core sequence and the 5′ end nontarget universal sequence. The poly(dI) linker prevents annealing of the 5′ end nontarget sequence to the template and facilitates primer hybridization at the 3′ end to the target sequence at specific temperatures, resulting in a dramatic improvement of annealing specificity [24].

In order to further improve the specificity, the universal primer (UP primer) of suppression PCR was used, which takes advantage of the priority of intrachain annealing than interchain ones. When there are inverted repeat sequences in both ends of PCR products (i.e., UP primer), the ends of the nontarget individual DNA strands will form “panhandle” structures (stem-loop structures) following every denaturation step. These structures would affect the bond of the primers and template, are more stable than the primer-template hybrid, and therefore will suppress exponential amplification [22, 25]. While a distal gene-specific primer extends a DNA strand through the UP primer site, the extension product will contain the UP primer sequence only on one end and thus cannot form the “panhandle” structure. PCR amplification can then proceed normally [25].

The following are the summaries of primer design of this new method.

- The binding sites of the target region in the unknown regions were created by the degenerate primers (LAD) of hiTAIL-PCR. However, the 3′ end specific gene primer of original ACP primer was replaced by the LAD primer of hiTAIL-PCR.
- The specificity of PCR amplification was improved by the suppression PCR through the introduction of UP primer. And it has got rid of the need of digestion and ligation by this. UP primer acted as the adapter primer of suppression PCR and the original 5′ end of ACP primer. The different LAD primers shared a common sequence in the 5′ half, that is, UP primer.
- The binding specificity of the primer with the template was controlled by ACP primers which consisted of UP primer at the 5′ end, intermediate [poly(dI)] linker, and LAD primers of hiTAIL-PCR at the 3′ end.
- The conserved primers of RNase H and PPT motif of RTNs replaced the gene-specific primers in the hiTAIL-PCR.
- The proportion of nontarget products was reduced by increasing the dilution ratio of the template in the next PCR amplification.

### 3.2. General Outline of the Technique

In total, three rounds of nested PCRs were employed in this study to improve the specificity. Firstly, the reaction was conducted in order to facilitate the RNase H1 primer to hybridize to the conserved RNase H domain of the template under the stringent conditions. For the ACP primer, 3′ end degenerate primer could anneal to the template and 5′ end could not anneal at the high annealing temperature. Therefore, ACP primer formed a vesicular structure due to the hybridization failure of low annealing [poly(dI)] to the template in this condition.

**Figure 1:**
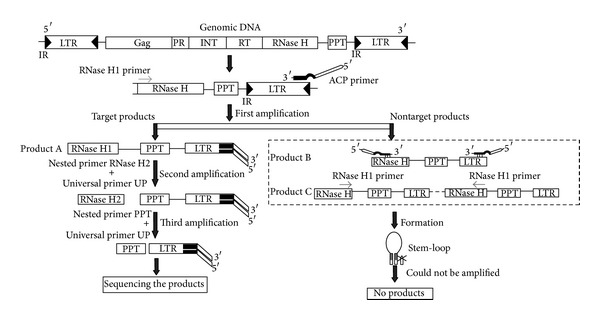
The schematic of the new method to isolate the LTR sequence of retrotransposons.

In fact, three kinds of different concentrations of PCR products were obtained after the first step, as shown in Figure 1. Product A is amplified from the combinations of RNase H1 primer and different ACP-LAD primers. Product B is just from ACP-LAD primer by itself, while Product C only resulted from RNase H1 primer. Among these products, Product A is the exact target products. Product C also seems as one of the target products in some extent, but it is very few and nearly could not be amplified due to the long distance of two RTNs. Product B is not the target products. But Product B only with ACP-LAD primer would not be amplified because they could not hybridize to the template under the high annealing temperature. In addition, nontarget products from nonspecific priming by the ACP-LAD primer alone, if any, are diluted and cannot be amplified in the following PCRs using the nested specific primers. Therefore, the target core sequences rise up and nontarget products are very few.

After 5 cycles of linear amplification of target sequences primed by RNase H1 primer and different ACP-LAD primer, which increases the copy numbers of the target molecules, a single cycle with a low annealing temperature (45°C) is carried out. The higher degree of the primer degeneracy and the low annealing temperature allow the LAD primer to bind to the target sequence with a higher probability, thus more target LTR sequence regions were efficiently created [23]. Then, the target sequences are amplified steadily at moderate annealing temperature (55°C) to increase their amounts in the following steps.

Then, the second round of PCR was followed with the 1000 times diluted PCR products as the template and amplified with the nested RNase H 2 primer and UP primer. The nonspecific products primed only by UP primer tend to form a stem-loop structure due to their complementary ends, which suppresses their amplification in the next amplification [25]. Thus, it decreased the nonspecific products. On the other hand, new nontarget products by UP primer alone cannot be generated and amplified to visible levels from such diluted (approximately 1000-fold) templates in the second PCR.

At last, the diluted PCR products of the second round were amplified for another nested PCR with PPT primer and UP primer. The specific products were obtained through this hierarchical PCR reaction and nontarget products were farthest suppressed.

### 3.3. Isolation of Tree Peony LTR Sequences

**Figure 2:**
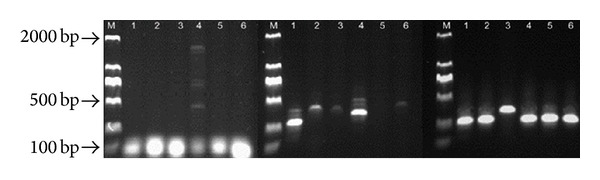
Amplification results from three rounds of PCR in Luoyanghong using the new method. It represented three rounds of PCR results from left to right. M, DL 2000 marker; 1–6, ACP primer 1–6.

The LTR sequences of tree peony RTNs were isolated with the newly developed method in this study. The representative tree peony cultivar in China, “Luoyanghong” (*Paeonia suffruticosa*), was used to perform the nested PCR. The amplification results of three rounds of PCR were shown in Figure 2. A smear of bands was usually showed by RNase H1 and one of the ACP-LAD primers in the first round of PCR (Figure 2), which contained a large number of randomly amplified products which resulted from tree peony genomic DNA. In some cases, the first round of amplified products were not detectable on the agarose gels (data were not shown), but target products could still be obtained in the next PCR. In some circumstances, the second amplification would also produce DNA smears. But the differential shift between the first and second products on agarose gels is a good indicator of the product specificity. In this study, the products resulting from the second round of PCR have some extent of specificity (Figure 2). When the third round of PCR was conducted, the specific products as a specific strong fragment were produced, which indicated the success.

A total of 22 independent clones were randomly selected and sequenced. After the exclusion of repeated sequences, 19 sequences possessed the expected PPT primer and UP primer, giving an average success rate of 86.36%. These sequences have been deposited in GenBank, and the accession numbers are from GenBank: KC519444 to GenBank: KC519464. Few duplicates were obtained, and so it is likely that many more new LTRs of RTNs could be obtained by the characterization of more subclones. This is much higher than that (50%–70%) of the original TAIL-PCR procedure [23]. All six LAD primers worked well in our tests.

Putative LTR sequence was firstly identified using TEClass [30] which is a software to identify different type of RTNs by universal structural features shared by LTRs-RTNs. And the characteristics of internal RTNs regions were also compared to the known structures of RTNs; that is, the polypurine tract site with the conserved AGGGGGAG motif [31] is located immediately upstream of the right LTR [32].

**Figure 3:**
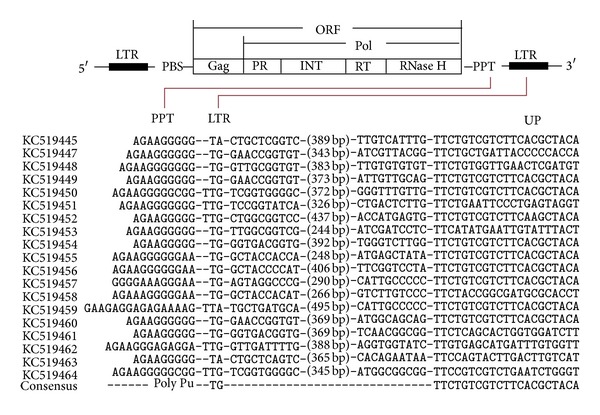
Alignment of nucleotide sequences of polypurine tracts (PPT) and 3′-LTR terminal sequences. Intervening sequence is of the size indicated in brackets.

These sequences were aligned using Lasergene 8.0 and the alignment results were shown in Figure 3. A continuous GA nucleotide motif (AGGGGG), that is, highly conserved in PPT motif, was observed in all 19 sequences, and it was followed by the TG/TA sequence which is the indication of the beginning of LTR sequences. However, not all identified LTRs began with the canonical sequences “TG,” which has also been observed in the study of Galindo et al. [15]. Although these regions differed in length (9–16 bp) and sequence characteristics, they all contained a conserved motif 5′-AGGGG-3′ [16]. While the distance of the predicted PPTs from the 3′-LTR start varied between 0 bp and 3 bp, the median of 1 bp indicated that they were mostly located at their expected positions as well [33]. These characteristics of PPT-LTR junction are also reported elsewhere [14, 15], supporting the reliability of the obtained LTR sequences herein. LTR sequences are highly variable both in sequence characteristics and in length. These sequences showed a similarity of 4.0–99.8% with an average of 18.9% in nucleotide sequences.

### 3.4. SSAP Analysis

**Figure 4:**
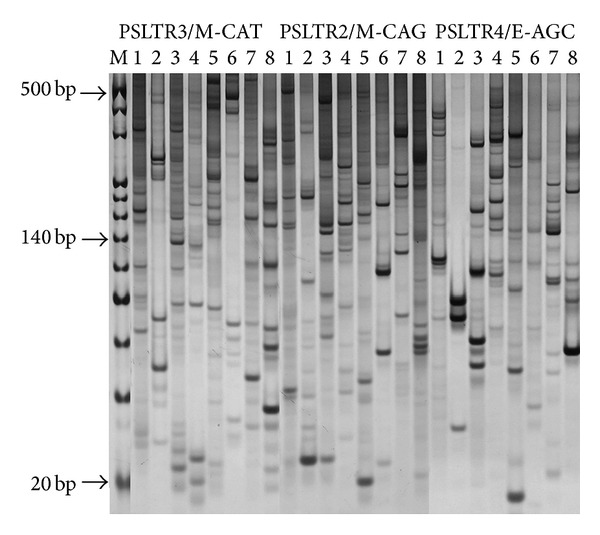
Comparison of SSAP profiles obtained with different retrotransposon and adapter primers. Each retrotransposon primer was used in combination with the* Mse* I or* EcoR* I adapter primer. Each set of eight lanes displays the reactions from the materials in Table 1 (from left to right). The primers of PSLTR 2, 3, and 4 are designed based on the sequence of GenBank: KC519450, GenBank: KC519454, and GenBank: KC519459, respectively.

SSAP is a RTNs-based marker system that utilizes sequence-specific RTNs-derived primers in combination with AFLP adapter primers. It is the most popular RTNs-based molecular marker method at present [6, 13, 21]. A number of LTRs were isolated in this study. In an attempt to evaluate the utility of these LTR sequences as molecular markers, we conducted a PCR survey to detect the polymorphic bands in a set of diverse tree peony genotypes which include different tree peony species, cultivar groups, colors, and flower forms (Table 1). The results showed that primers designed on these LTRs allowed to evidence highly polymorphic SSAP fingerprints in* Paeonia*. Example of SSAP silver staining result was shown in Figure 4. A high quality profile displays abundant, intense bands against a low background. A large number of clear bands with a high percentage of polymorphisms were produced which were corresponding to the diversities of the diverse materials. These results showed that the SSAP marker system based on the isolation of tree peony LTR sequences could be used effectively for exploring polymorphism among tree peony varieties.

### 3.5. The Characteristics and Advantage of the New Method

The method reported herein has several advantages over previous studies on the isolation of LTR sequences [14, 15, 18, 19].

#### 3.5.1. Low False Positive

Kalendar et al. [10] designed iPBS primers and used them not only as molecular markers but also as the isolation method of RTN-LTR sequences. They could obtain many sequences at one time; however, a majority of these sequences may lack typical or complete RTN-LTR sequences [10, 34]. The high false positive is a main obstacle of TAIL-PCR due to its short random primer (10~13 bp) which resulted in the nonspecific binding with template in low annealing temperature. The new method employed the improved hiTAIL-PCR [23] which used the longer LAD primer and in combination with ACP system which have a [poly(dI)] linker [24]. So it makes the modified ACP primers could specifically anneal to the template in a high annealing temperature (60°C) during the initial step.

#### 3.5.2. Easy Application

This method is just based on PCR and agarose gel technique. It directly aimed at the LTR region and has removed the necessity of the procedure of hybridization, washing, screening, and so forth, which is used in the method of Pearce et al. [14]. Compared with other isolation methods, the PCR-RAGE method [20] requires the enzyme digestion and other tedious procedures; the SiteFinding-PCR method [18] also needs enzyme digestion and another six gene-specific primers with two SiteFinder primers. The new method did not need enzyme digestion or adapter ligation. It just needs the PCR, so it is very easy.

#### 3.5.3. High Repeatability

The method of Pearce et al. [14] needs several times of hybridization and washing. Because the efficiency of hybridization and washing is largely different each time, so the results of different reiteration would be varied which affected the repeatability. The method of Zhao et al. [18] needs to design six gene-specific primers, so different gene-specific primers had to be designed when every new LTR sequence was isolated. This method just employs three nested primers and one special designing primer which randomly combined in the target region and has simplified the heavy and complicated steps of hiTAIL-PCR to reduce the chance of error imported; therefore the repeatability is highly improved due to the high annealing temperature. The specific primer in the original ACP system has been replaced by the LAD primers.

#### 3.5.4. Rapid and Economical

All reactions of this method could be completed only in one day. The target sequence could be obtained in short time and does not need to exclude the false positive. Otherwise, the additional conductions were needed to identify the reliability of candidate sequence in other methods [14, 20]. This method could isolate enough target sequences one time and could be applied to another amplification of unknown sequence in the vicinity of any known sequence except for the isolation of LTR sequences.

## 4. Conclusion

A new PCR method to isolate the LTR sequences of RTNs was developed in tree peony. This method combined the advantages of hiTAIL-PCR, suppression PCR, and annealing control primer (ACP) methods and reduced the nonspecific amplification by random degenerate primers in hiTAIL-PCR by suppression effects of suppression PCR and high annealing temperature increasing of primers. This method is rapid, economical, and cost- and time-saving which could be easily used to isolate LTR sequences of RTNs in other plants.

## Figures and Tables

**Table 1:** Characteristics of tree peony genotypes used for SSAP analysis.

Code	Cultivar (species)	Flower form	Cultivar group	Color
1	Luoyanghong	Rose form	Zhongyuan	Purple
2	Yinhongqiaoyu	Chrysanthemum form	Zhongyuan	Red
3	Lantianyu	Crown form	Zhongyuan	Blue
4	Doulv	Globular form	Zhongyuan	Green
5	Er Qiao	Rose form	Zhongyuan	Bicolor
6	Fengdan	Single form	Jiangnan	White
7	*P*. *rockii *	Single form	The wild	White
8	*P*. *ludlowii *	Single form	The wild	Yellow

**Table 2:** The components of three PCR rounds, respectively.

Component	First PCR amplification	Second PCR amplification	Third PCR amplification
Template DNA	50–100 ng	1000 times dilution of PCR products from the first PCR	1000 times dilution of PCR products from the second PCR
Buffer (TaKaRa)	1x	1x	1x
Mg^2+^	2.5 mmol·L^−1^	2.5 mmol·L^−1^	2.5 mmol·L^−1^
dNTPs	0.2 mmol·L^−1^	0.2 mmol·L^−1^	0.2 mmol·L^−1^
RNase H1 primer	0.4 *μ*mol·L^−1^	—	—
RNase H2 primer	—	0.4 *μ*mol·L^−1^	—
ACP primer	0.8 *μ*mol·L^−1^	—	—
PPT primer	—	—	0.4 *μ*mol·L^−1^
Universal primer UP	—	0.4 *μ*mol*·*L^−1^	0.4 *μ*mol·L^−1^
Taq polymerase	1.0 U	1.0 U	1.0 U
Total volume	10 *μ*L	20 *μ*L	20 *μ*L

**Table 3:** The programme parameters of three rounds of PCR.

Phase	Step	Cycle conditions	Cycles
First PCR round	1	94°C for 5 min	1
	2	94°C for 50 s, 60°C for 1 min, and 72°C for 2 min	5
	3	94°C for 50 s, 45°C for 30 s, and 72°C for 2 min	1
	4	94°C for 50 s, 55°C for 30 s, and 72°C for 2 min	25
	5	72°C for 8 min	1

Second PCR round	1	94°C for 5 min	1
	2	94°C for 50 s, 55°C for 30 s, and 72°C for 2 min	30
	3	72°C for 10 min	1

Third PCR round	1	94°C for 5 min	1
	2	94°C for 1 min, 55°C for 30 s, and 72°C for 2 min	30
	3	72°C for 10 min	1

**Table 4:** The sequences of primers used in this study.

Primers		Sequence
Nested primer	RNase H1	MGNACNAARCAYATHGA
	RNase H2	GCNGAYATNYTNACNAA

ACP primer	ACP 1	**TGTAGCGTGAAGACGACAGAA **IIIII VNVNNNGGAA
	ACP 2	**TGTAGCGTGAAGACGACAGAA** IIIII BNBNNNGGTT
	ACP 3	**TGTAGCGTGAAGACGACAGAA** IIIII HNVNNNCCAC
	ACP 4	**TGTAGCGTGAAGACGACAGAA** IIIII CAATGGCTACCAC
	ACP 5	**TGTAGCGTGAAGACGACAGAA** IIIII VVNVNNNCCAA
	ACP 6	**TGTAGCGTGAAGACGACAGAA** IIIII BDNBNNNCGGT

UP primer	UP	**TGTAGCGTGAAGACGACAGAA**

PPT primer		RRRRRRRRRRRRRRRR

Note: the bold letters represent the nontarget universal sequences. I represent deoxyinosine. The meaning of degenerate base: B (C/G/T), D (A/G/T), H (A/C/T), R (A/G), N (A/G/C/T), and V (A/C/G).
